# Hepatocyte growth factor and basic fibroblast growth factor regulate atrial fibrosis in patients with atrial fibrillation and rheumatic heart disease via the mitogen-activated protein kinase signaling pathway

**DOI:** 10.3892/etm.2013.1274

**Published:** 2013-08-28

**Authors:** MINGJIANG LI, XIN YI, LELE MA, YANLI ZHOU

**Affiliations:** Department of Cardiology, Renmin Hospital of Wuhan University, Wuhan, Hubei 430060, P.R. China

**Keywords:** basic fibroblast growth factor, hepatocyte growth factor, mitogen-activated protein kinase signaling pathway, atrial fibrosis, rheumatic heart disease

## Abstract

The aim of this study was to investigate the interrelation between basic fibroblast growth factor (bFGF), hepatocyte growth factor (HGF) and atrial fibrosis in patients with atrial fibrillation (AF) and rheumatic heart disease (RHD), and to explore the possible molecular mechanisms underlying this interrelation. Twenty patients with RHD who were scheduled for valve replacement were divided into two groups, comprising 10 cases with AF and 10 cases with sinus rhythm (SR). Clinical data were collected and a small sample of aseptic left atrial appendage was collected by the surgeon. Hematoxylin and eosin (H&E) and Masson’s trichrome-stained sections were used to evaluate the cross-sectional area and level of fibrosis, respectively. The expression levels of bFGF and HGF were assessed using immunohistochemistry. The phosphorylation levels of mitogen-activated protein kinase/extracellular signal-regulated kinase 1/2 (MEK1/2), c-Jun N-terminal kinase 1/2 (JNK1/2), extracellular signal-regulated kinase 1/2 (ERK1/2) and p38 in atrial tissue were measured using western blotting. Compared with the SR group, myocardial cell diameter was significantly expanded and there was increased collagen deposition in the AF group (P<0.05). The distribution of bFGF in the AF group was significantly higher than that in the SR group (P<0.05); however, HGF levels were significantly lower in the AF group (P<0.05). The phosphorylation levels of MEK1/2, ERK1/2, JNK1/2 and p38 in the AF group were significantly higher than those in the SR group (P<0.05). The results indicated that bFGF may promote the development of atrial fibrosis, while HGF may function in an opposite manner in patients with AF and RHD. The mitogen-activated protein kinase (MAPK) signaling pathway may be the molecular basis for these roles in atrial fibrosis.

## Introduction

Atrial fibrillation (AF) is one of the most frequently occurring clinical diseases and is a common clinical manifestation of rheumatic heart disease (RHD), hypertension, coronary heart disease, congenital heart disease, cardiomyopathy, pericardial diseases and other cardiovascular disease. AF presents a serious threat to the health of the individual. In addition, mural thrombus, induced by AF, may lead to serious cardiovascular events with high rates of morbidity and mortality (1). Previous studies have shown that radiofrequency catheter ablation may be used to narrow the left atrial diameter (LAD) and significantly improve the left ventricular ejection fraction (LVEF) and cardiac function in patients with AF, indicating that the remodeling of atrial structure is crucial in the development of AF (2–5). Therefore, blocking the remodeling of the atrial structure maybe an improved method of preventing the development of AF. Cardiac fibrosis, which is a common pathology of numerous cardiovascular diseases, has become of particular interest in recent years. This is due to a desire to provide a theoretical basis for the development of novel targets for anti-fibrotic therapies.

Clinical and non-clinical studies have demonstrated that atrial fibrosis is the most prominent manifestation of atrial structural remodeling in patients with AF (6–8). The electrical conductivity heterogeneity in atrial fibrosis facilitates the occurrence and maintenance of AF (9–12). One of the primary factors leading to fibrosis is an imbalance between fibrogenic and antifibrotic cell growth factors. Basic fibroblast growth factor (bFGF) is a fibrogenic cell growth factor, while hepatocyte growth factor (HGF) has been identified to be a unique antifibrotic cell growth factor.

In addition to promoting cell differentiation, mitosis, tumor occurrence and metastasis, HGF is also involved in antifibrotic processes and exerts a variety of biological effects (13–14). Iwata *et al* (15) demonstrated that a low degree of myocardial fibrosis was present with lower levels of HGF in the myocardial tissue of rats and that myocardial collagen expression and distribution were significantly reduced following HGF overexpression (15). HGF is the ligand of c-Met, which has a domain with protein tyrosine kinase activity (16). c-Met transduces signals from the extracellular matrix into the cytoplasm by binding with HGF/HGF ligand to regulate a number of physiological processes, including cell proliferation, scattering, morphogenesis and survival. Ligand binding at the extracellular domain induces the autophosphorylation of c-Met in its intracellular domain, which provides docking sites for downstream signaling molecules (17). Following activation by its ligand, c-Met interacts with the phosphoinositide (PI) 3-kinase subunit phosphoinositide-3-kinase regulatory subunit 1 (PIK3R1), phospholipase C γ 1 (PLCG1), SRC, growth factor receptor-bound protein 2 (GRB2), signal transducer and activator of transcription 3 (STAT3) or the adapter GRB2-associated-binding protein 1 (GAB1), which is necessary for c-Met to activate a number of signaling cascades, including RAS-extracellular signal-regulated kinase (ERK), PI3-kinase-AKT and phospholipase C γ-protein kinase C (PLCγ-PKC). The RAS-ERK activation is involved with morphogenetic effects, while PI3K/AKT coordinates pro-survival effects (18). During embryonic development, c-Met signaling is important in gastrulation, development and migration of muscles and neuronal precursors, angiogenesis and kidney formation. In adults, it participates in wound healing, as well as organ regeneration and tissue remodeling (19). However, its role in atrial fibrosis has not yet been clarified. A previous study demonstrated that bFGF-fibroblast growth factor receptor (FGFR)-heparan sulfate proteoglycan (HSPG) complexes were able to activate the mitogen-activated protein kinase (MAPK; ERK1/2) signaling pathway, thereby activating cardiac fibroblasts and leading to collagen deposition, decreased degradation, disorder of metabolic balance and, ultimately, fibrosis (20). Therefore, this study aimed to investigate the interrelation of bFGF, HGF and the MAPK signaling pathway with atrial fibrosis in patients with AF and RHD. The results indicate that bFGF is able to promote the development of atrial fibrosis, while HGF functions in an opposite manner in patients with AF and RHD. The MAPK signaling pathway may be the molecular basis for these roles in atrial fibrosis.

## Patients and methods

### Study population

Twenty patients with RHD who underwent valve replacement were included as the study subjects. The patients were aged between 30 and 70 years and had heart function grades ranging from I to III. The exclusion criteria were: infective endocarditis, hyperthyroidism, serious liver, kidney or lung dysfunction, malignant tumor, coronary athero-sclerotic heart disease and chronic pulmonary heart disease. The patients were divided into two groups, with 10 patients in the sinus rhythm (SR) group and 10 in the AF group. The study was approved by the Ethics Committee of Renmin Hospital of Wuhan University (Wuhan, China) and all patients provided written informed consent.

### Human myocardium samples

Samples were collected from the right atrium of 20 patients with RHD who underwent valve replacement. Written informed consent was obtained from the family of prospective donors and the patient. The samples were obtained according to the regulations of the Cardiovascular Research Institute of Wuhan University.

### Materials

Primary antibodies against p38 (cat. no. 9212), mitogen-activated protein kinase/extracellular signal-regulated kinase 1/2 (MEK1/2; cat. no. 9122), c-Jun N-terminal kinase 1/2 (JNK1/2; cat. no. 9258), phospho-MEK1/2Ser217/221 (cat. no. 9154), phospho-JNK1/2 (cat. no. 4668), ERK1/2 (cat. no. 4695), phospho-ERK1/2Thr202/Thr204 (cat. no. 4370) and phospho-p38Thr180/Thr182 (cat. no. 4511) were purchased from Cell Signaling Technology, Inc. (Danvers, MA, USA). Antibodies against HGF (ab10678) were purchased from Abcam Ltd. (Cambridge, UK). Connective tissue growth factor (CTGF; sc-73869) and glyceraldehyde-3-phosphate dehydrogenase (GAPDH; MB001) antibodies were obtained from Santa Cruz Biotechnology, Inc. (Santa Cruz, CA, USA) and Bioworld Technology Inc. (Minneapolis, MN, USA) respectively. The bicinchoninic acid (BCA) protein assay kit was purchased from Pierce Biotechnology (Rockford, IL, USA).

### Histological analysis

During surgery, ~200 mg of the right atrial myocardium was collected prior to extracorporeal circulation being established. This sample of myocardium was subsequently divided into two parts; one part was rapidly placed in a liquid nitrogen jar and immediately transferred to a −80°C refrigerator and the remaining part was immediately washed with saline solution and fixed with 10% neutral buffered formalin. Following this, several sections (4–5 *μ*m thick) were prepared and the hematoxylin and eosin (H&E)-stained sections were used to determine the cross-sectional area of the myocytes. Evidence of interstitial and perivascular collagen deposition was visualized using Masson’s trichrome staining and then high-magnification light micrographs were captured using light microscopy. Collagen volume (%) was measured using an image quantitative digital analysis system (Image-Pro Plus 6.0, Media Cybernetics, Inc., Rockville, MD, USA).

### Immunohistochemistry

Using the EnVision^™^ two-step method, the tissue specimens were fixed in formaldehyde solution, embedded in paraffin and sliced. The paraffin was then removed. HGF and bFGF antibodies were added at a concentration of 1:100, prior to the specimens being incubated for ~60 min at room temperature and rinsed three times in phosphate-buffered saline (PBS). EnVision^™^ (50 *μ*l) reagent was added to each section and the sections were subsequently incubated for ~60 min at room temperature, flushed with PBS, stained with 3,3’-diaminobenzidine (DAB), counterstained with hematoxylin and placed on a neutral gum mount. The appearance of red or brownish yellow granules in the cytoplasm indicated a positive result. Computer image analysis was used to determine the density of the positively stained area and for relative quantitative analysis.

### Western blotting

A total of 50 *μ*g protein was extracted from the myocardial tissue, lysed in radio-immunoprecipitation assay (RIPA) lysis buffer and used for sodium dodecyl sulfate-polyacrylamide gel electrophoresis (SDS-PAGE). The proteins were then transferred to nitrocellulose membranes and blocked with 5% non-fat dry milk in Tris-buffered saline (TBS) for 90 min at room temperature. Following this, the membranes were probed with various primary antibodies overnight. The next day, the membranes were washed with 1X TBS and Tween 20 (TBST) and incubated for 1 h with horseradish peroxidase-labeled mouse anti-rabbit antibody (1:2,000) and anti-avidin antibodies (1:1,000) in double anti-TBST fluid. Following the membrane being washed three times, the film was placed in 10 ml LumiGLO^®^ solution for 1 min. After being developed, the images were placed into an automatic image analyzer to determine the expression of the proteins and the reference gray-scale values. A monoclonal anti-GAPDH antibody was used separately as a loading control.

### Statistical analysis

The data are presented as the mean ± standard error of the mean. Comparisons between two groups were performed using an unpaired Student’s t-test. P<0.05 was considered to indicate a statistically significant difference.

## Results

### General clinical characteristics of the two groups of patients

All patients underwent preoperative routine testing of urine, stools, blood coagulation, blood biochemistry, chest X-ray, electrocardiography and ultrasonic cardiogram. The general clinical characteristics that were analyzed included age, gender, LVEF, LAD and AF duration. With regard to gender, age, New York classification of cardiac function (NYHA) and LVEF, the two groups were not significantly different (Table I). However, in the AF group, the LAD was significantly increased when compared with that of the SR group (P<0.05).

### Myocardial cell and fibrosis morphology

**Figure 1.**
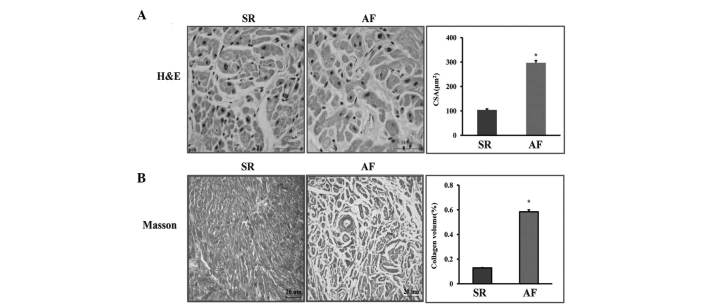
Histological analysis of the right atrium of patients with rheumatic heart disease (RHD). (A) Hematoxylin and eosin (H&E) staining in the right atrium of patients with RHD with sinus rhythm (SR) and atrial fibrillation (AF). Left, representative image; right, statistical results for the cell surface area (CSA). *P<0.05 vs. the SR group; magnification, ×400. (B) Masson’s trichrome staining in the right atrium of patients in the SR and AF groups. Left, representative image; right, quantification of the total collagen volume for the SR and AF groups. *P<0.05 vs. the SR group; magnification, ×400.

To investigate the role of AF in the morphology of patients with RHD, samples were collected from the right atrium of twenty patients with RHD who underwent valve replacement. H&E and Masson’s trichrome staining indicated that AF had an adverse effect on cardiac remodeling. From the H&E staining, it was observed that the myocardial cell diameter of the patients in the AF group was significantly expanded. Fibrosis was quantified by visualizing the total amount of collagen present in the interstitial spaces of the myocardial tissue and by determining the collagen volume. Interstitial fibrosis was observed in the SR group and the AF group; however, it was markedly increased in the AF group. The AF group showed a significant increase in total collagen volume compared with that in the SR group (P<0.05; Fig. 1).

### Effects of AF on bFGF and HGF

**Figure 2.**
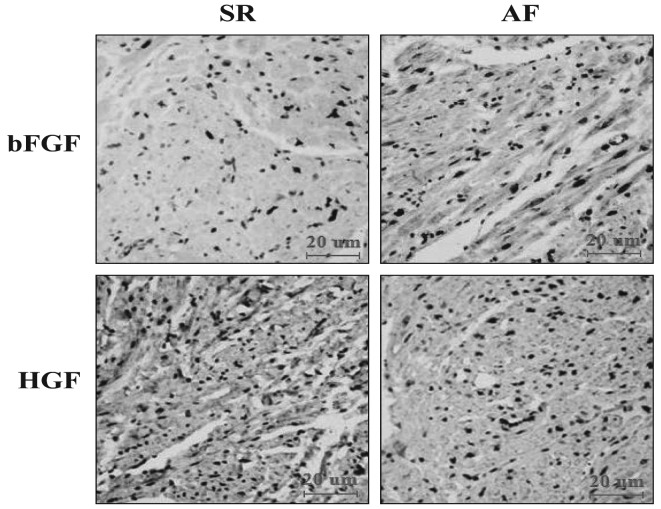
Expression of basic fibroblast growth factor (bFGF) and hepatocyte growth factor (HGF) in the right atrium of patients with rheumatic heart disease (RHD). Top, representative image of bFGF immunohistochemistry; bottom, representative image of HGF immunohistochemistry; magnification, ×400.

Immunohistochemical staining for bFGF and HGF was performed in the tissue sections in order to assess the expression levels of bFGF and HGF. The results showed that the intracellular distribution of small bFGF granules in the atrial myocytes of the SR group was lower than that in the AF group. By contrast, the levels of HGF were significantly lower in the AF group compared with those in the SR group (Fig. 2).

### Effects of AF on the MAPK signaling pathway

**Figure 3.**
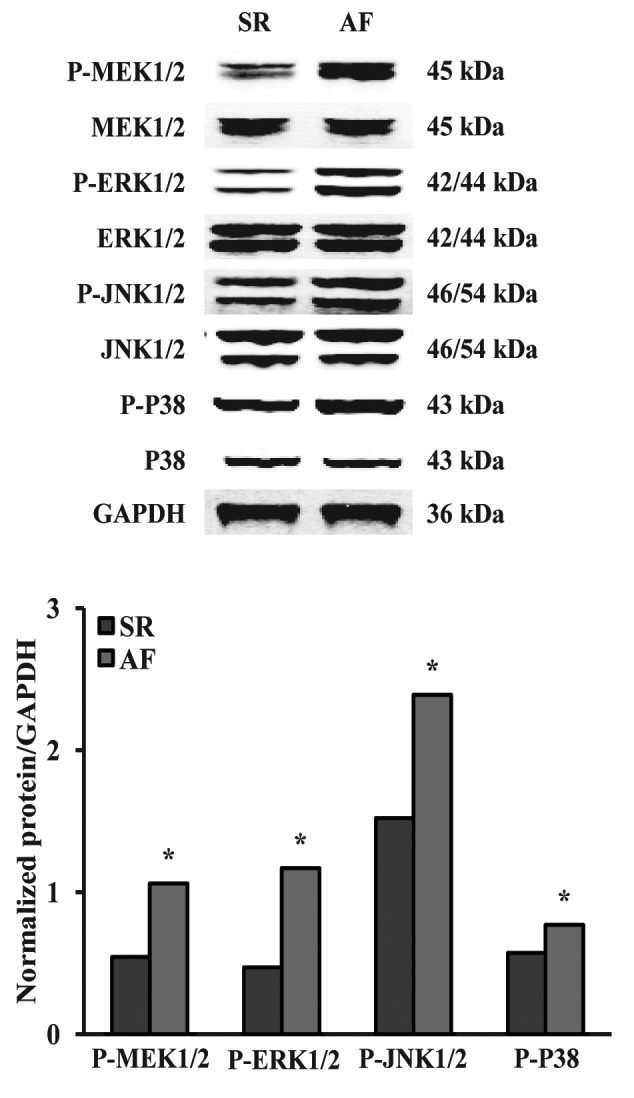
Effect of atrial fibrillation (AF) on the mitogen-activated protein kinase (MAPK) signaling pathway. Top, representative western blot analysis showing the phosphorylation (P) and total protein expression of MAPK/extracellular signal-regulated kinase 1/2 (MEK1/2), extracellular signal-regulated kinase 1/2 (ERK1/2), c-Jun N-terminal kinase 1/2 (JNK1/2) and p38. Bottom, quantitative results of the MEK1/2, ERK1/2, JNK1/2 and p38 phosphorylation levels in patients with rheumatic heart disease with sinus rhythm (SR) and AF, respectively. *P<0.05 vs. SR group. GAPDH, glyceraldehyde-3-phosphate dehydrogenase.

To explore the molecular mechanisms underlying the increased bFGF levels and decreased HGF levels in the AF group, we investigated the MAPK signaling pathway. It was observed that the phosphorylation levels of MEK1/2, ERK1/2, p38 and JNK1/2 were significantly increased in the AF group compared with those in the SR group (Fig. 3).

## Discussion

The most important observation in this study was that myocardial cell diameter and levels of fibrosis were significantly increased in patients with AF. Immunohistochemical staining showed that levels of bFGF were increased, while levels of HGF were reduced in the patients with AF compared with those in the SR group. Further experiments showed that the phosphorylation level of components of the MAPK pathway was increased markedly in the AF group. To the best of our knowledge, these results are the first direct indication that the expression levels of bFGF and HGF, which are closely interrelated with fibrosis, are regulated by the MAPK pathway in patients with AF.

The pathogenesis of AF is complex and has not been completely elucidated. One recognized theory is that the occurrence and maintenance of AF are closely associated with atrial remodeling, including electrical and structural remodeling, and that atrial fibrosis is the most important part of the structural remodeling (21). An animal model study of AF demonstrated the occurrence of atrial collagen hyperplasia and AF from which the recovery of natural SR was rare. In addition, while collagen hyperplasia and accumulation in the interstitial cells affected the entire mechanics of the atrial systolic and diastolic function, it also caused local electro-cardiac heterogeneity in conduction, resulting in arrhythmias and, in particular, AF (22). In the present study, we observed that the atrial myocyte diameter and levels of fibrosis were increased in patients with RHD and AF, using H&E and Masson’s trichrome staining, respectively. In the SR group, only a few collagen fibers were observed and there was little interstitial fibrosis. The results of this study were consistent with a previous study, which revealed that rheumatic mitral valve disease and atrial structural remodeling were closely associated with the occurrence of AF (23).

bFGF is a member of the FGF family. FGF family members bind heparin and possess broad mitogenic and angiogenic activities (24). bFGF has been implicated in diverse biological processes, such as limb and nervous system development (25), wound healing (26) and tumor growth (27). A previous study in an animal model demonstrated that bFGF was important in continuous hemodynamic-overload stimulation-induced myocardial cell hypertrophy, myocardial fibrosis and myocardial collagen hyperplasia (28). Our result showed that bFGF was more diffusely distributed in the myocardial cells of the patients with AF than in those in the SR group, .

HGF, another cell growth factor, is secreted by mesenchymal cells and acts as a multi-functional cytokine on cells of mainly epithelial origin. It regulates cell growth, motility and morphogenesis by activating a tyrosine kinase signaling cascade subsequent to binding to the proto-oncogenic c-Met receptor. The ability of HGF to stimulate mitogenesis, cell motility and matrix invasion makes it pivotal in angiogenesis, tumorigenesis and tissue regeneration. Taniyama *et al* (29) studied hamster cardiomyopathy and observed that, in lesions of the myocardium, the levels of HGF mRNA and protein expression were reduced. Furthermore, myocardial fibrosis and changes in cell shape were also observed. The results of the immunohistochemical staining in our study showed that HGF was highly expressed in the cytoplasm of the atrial myocytes of the SR group, whereas the expression was significantly lower in the AF group. Inoue *et al* (30) revealed that HGF counteracted transforming growth factor β1 (TGFβ1) through the attenuation of CTGF induction and prevented renal fibrogenesis in five out of six nephrectomized mice. Jun *et al* (31) showed that HGF/c-Met was able to enhance the proliferation and suppress the expression of the fibrosis marker α-smooth muscle actin (α-SMA) in ARPE-19 cells.

c-Met is a receptor tyrosine kinase that transduces signals from the extracellular matrix into the cytoplasm by binding to HGF/HGF ligand. It regulates a number of physiological processes, including proliferation, scattering, morphogenesis and survival. Ligand binding at the cell surface induces the autophosphorylation of Met in its intracellular domain, which provides docking sites for downstream signaling molecules. Following activation by its ligand, c-Met interacts with the PI3-kinase subunit PIK3R1, PLCG1, SRC, GRB2, STAT3 or the adapter GAB1. The recruitment of these downstream effectors by Met leads to the activation of numerous signaling cascades, including RAS-MAPK kinase (MAPKK)-MAPKs (ERK/p38/JNK). The activation of RAS-MAPKK-MAPK is associated with the morphogenetic effects. Previous studies (32–34) have shown that MAPKs are important in the process of fibrosis. The results in present study indicated that the MAPK signaling pathway showed a significantly increased level of activation in the AF group, and that the phosphorylation levels of MEK1/2, ERK1/2, p38 and JNK1/2 were notably increased in the AF group, compared with those in the SR group. Lu *et al* (35) indicated that the phosphorylation level of ERK1/2 was significantly lower in claudin-7 transfected cells than control cells following HGF treatment. In addition, Cohen *et al* (36) demonstrated that oncostatin M (OSM)-induced HGF secretion was inhibited by PD-98059 (a specific pharmacological inhibitor of ERK1/2), SB-203580 (a p38 MAPK inhibitor) and SP-600125 (a JNK inhibitor) by 70, 82 and 100%, respectively. Yang *et al* (37) showed that bFGF was able to induce neuronal differentiation of mouse bone marrow stromal cells via FGFR-1, MAPK/ERK and activator protein 1 (AP-1). bFGF has also been demonstrated to activate the MAPK and nuclear factor κB (NFκB) pathways to control the production of matrix metalloproteinase-13 in human adult articular chondrocytes (38).

In conclusion, bFGF may promote the development of atrial fibrosis, while HGF may function in an opposite manner in patients with RHD and AF. The MAPK signaling pathway may be the molecular basis for these effects in atrial fibrosis.

## Figures and Tables

**Table I.** General clinical characteristics of the study population.

Characteristics	SR (n=10)	AF (n=10)
Gender (male/female)	4/6	5/5
Age (years)	46.01±10.38	49.51±11.04
AF duration (months)	-	10.51±2.04
NYHA (II/III)	3/7	4/6
LAD (mm)	42.41±7.31	57.23±12.30^a^
LVEF (%)	62.01±9.38	58.21±10.80

Age, LAD and LVEF are presented as the mean ± standard error of the mean. Gender and grade of heart function were compared using Fisher’s exact probability method;

a P<0.05 compared with the SR group. SR, sinus rhythm; AF, atrial fibrillation; LAD, left atrial diameter; LVEF, left ventricular ejection fraction; NYHA, New York heart function classification.
